# FCS–MPC with Nonlinear Control Applied to a Multicell AFE Rectifier †

**DOI:** 10.3390/s22114100

**Published:** 2022-05-28

**Authors:** Eduardo Espinosa, José Espinoza, Pedro Melín, Jaime Rohten, Marco Rivera, Javier Muñoz

**Affiliations:** 1 Department of Electrical Engineering, Faculty of Engineering, Universidad Católica de la Santísima Concepción, Concepción 4090541, Chile; 2 Department of Electrical Engineering, Universidad de Concepción, Concepción 4070386, Chile; jose.espinoza@udec.cl; 3 Department of Electrical and Electronic Engineering, Universidad del Bío-Bío, Concepción 4051381, Chile; pemelin@ubiobio.cl (P.M.); jaimerohten@ubiobio.cl (J.R.); 4 Department of Electrical Engineering, Universidad de Talca, Curicó 3340000, Chile; marcoriv@utalca.cl (M.R.); jamunoz@utalca.cl (J.M.)

**Keywords:** AC-DC power converters, nonlinear control systems, predictive control, total harmonic distortion reduction

## Abstract

The use of controlled power converters has been extended for high power applications, stacking off-the-shelve semiconductors, and allowing the implementation of, among others, AC drives for medium voltages of 2.3 kV to 13.8 kV. For AC drives based on power cells assembled with three-phase diode rectifiers and cascaded H-bridge inverters, a sophisticated input multipulse transformer is required to reduce the grid voltage, provide isolation among the power cells, and compensate for low-frequency current harmonics generated by the diode-based rectifiers. However, this input multipulse transformer is bulky, heavy, and expensive and must be designed according to the number of power cells, not allowing total modularity of the AC drives based on cascade H-bridges. This study proposes and evaluates a control strategy based on a finite control set-model predictive control that emulates the harmonic cancellation performed by an input multipulse transformer in a cascade H-bridge topology. Hence, the proposed method requires conventional input transformers and replaces the three-phase diode rectifiers. As a result, greater modularity than the conventional multicell converter and improved AC overall input current with a THD as low as 2% with a unitary displacement power factor are achieved. In this case, each power cell manages its own DC voltage using a nonlinear control strategy, ensuring stable system operation for passive and regenerative loads. The experimental tests demonstrated the correct performance of the proposed scheme.

## 1. Introduction

Multilevel converters are widely used in AC drive applications for feeding AC motors in the medium voltage range (up to 13.8 kV) and are characterized by low-voltage semiconductors [1,2,3,4,5,6]. The multilevel converter features are (i) load AC voltage with low distortion and low dv/dt, (ii) low switching frequency, and (iii) low common-mode voltage. The classic topologies are: neutral point clamped (NPC) [7], cascaded H-bridge (CHB) [8], and capacitor clamped [9].

CHB topologies connect several cells in series to the AC load side to increase the resulting AC voltage [9]. Each power cell is composed of (i) a rectifier stage, which is commonly implemented using a three-phase diode rectifier, (ii) a DC link stage based on an electrolytic capacitor array, and (iii) a single-phase voltage source inverter. Given the characteristics of a series connection of the power cells on the AC load side, isolated DC sources are required and provided using an input transformer with multiple secondaries. This allows a reduced voltage supply in the power cells, facilitating the use of components with low voltage ratings, such as semiconductor devices and DC capacitors. Furthermore, the multipulse transformer allows low-frequency current harmonics cancellation generated by diode rectifiers, obtaining an AC total input current with low THD [9].

Several strategies have been reported in the literature to improve the performance of the input multipulse transformer in multicell converters [10,11,12]. Similarly, the replacement of the three-phase diode rectifier with an AFE rectifier and the employment of modulation techniques designed to improve the overall current and control schemes are reported in [13,14,15], Table 1. However, despite these efforts, the topology still requires an input multipulse transformer, which is bulky, expensive, complicated to manufacture, and must be designed for a specific number of power cells and power, which avoids extending the modularity of the power cell up to the input transformer.

Model predictive control (MPC) is a control strategy used in power converters [16,17,18,19], which is classified into two categories: continuous control set MPC, and finite control set MPC (FCS–MPC). The FCS–MPC [20,21,22,23] uses the natural discrete operation of power converters and easily allows the inclusion of (i) the converter operation nonlinearities and constraints, (ii) the rapid response to reference changes, and (iii) it is not necessary for a modulation stage to apply the control action in the power converter.

On the other hand, the utilization of a nonlinear control approach in the DC voltage loop guarantees the stable operation of the AFE, even in the regenerative operating mode [24,25,26,27].

This work proposes to modify the traditional topology of the power cell for AC drives based on CHB. Indeed, the large input multipulse transformer (sized for the total power) is replaced by simple and smaller ones that are incorporated into the power cell (sized for the power of the power cell). In addition, it uses a master-slave control loop in each power cell to ensure the stable operation of the AFE rectifier. This approach overcomes the following drawbacks: (i) it replaces the multipulse input transformer with a simpler design (K-Factor = 3.0), (ii) it obtains AC grid currents with low THD (<2.0%) and unity displacement factor, and (iii) it ensures the stable operation of the AFE rectifier, including regeneration, as it uses a nonlinear control law for the DC voltage loop.

The paper is organized as follows. First, the proposed topology is shown in Section 2, following the overall input current **i***_g_^abc^*. THD minimization is presented in Section 3, which allows for the obtaining of a fixed harmonic spectrum in the input current **i***_si_^abc^* of each power cell. In addition, the multipulse input transformer is replaced by a conventional wye-to-wye transformer to extend the modularity of the power cell, leaving the task of harmonic minimization to the AFE current control loop. Thus, this allows a low THD in the AC main currents and ordinary input transformers instead of an input multipulse transformer. Then, in Section 4, a control scheme is presented. In each power cell, a master-slave control loop was analyzed. In the outer loop, a nonlinear control scheme is set to regulate the DC voltage of each cell, where the main feature of this option is the resulting stable operation of the topology over the entire operating region. On the other hand, the inner loop is the current FCS–MPC, based on the input current model for each power cell. Finally, Section 5 presents the experimental tests to support the proposed scheme.

**Table 1:** Summary of proposals on AC drive based on CHB topology–input AC side.

Proposed Changes on CHB Topology–Input AC Side	Papers
Modification input multipulse transformer	[10,11,12]
Replace three-phase diode rectifier	[13,14,15]
Replace three-phase diode rectifier and input multipulse transfomer	[28]

## 2. Topology

**Figure 1:**
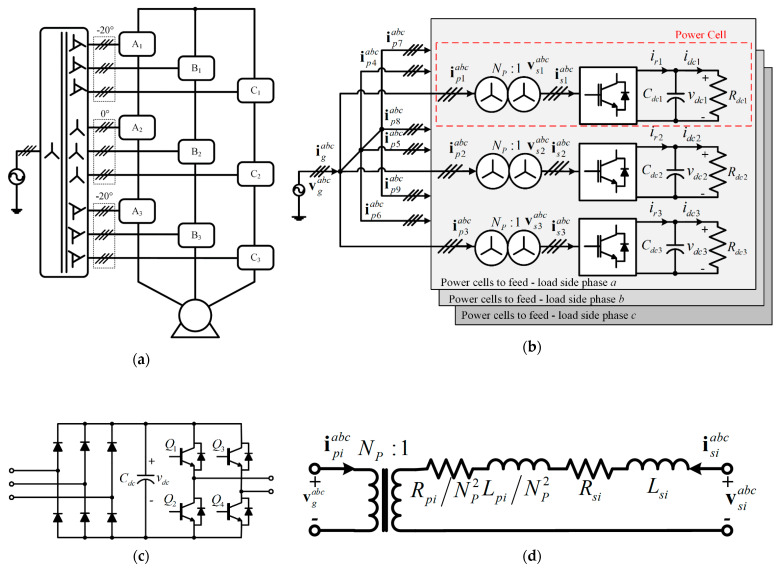
(**a**) AC drive based on CHB, (**b**) traditional power cell for AC drive based on CHB, (**c**) proposed topology, (**d**) wye–wye input transformer model referred to the secondary winding.

In multicell rectifiers, the multipulse transformer is the one that performs the task of canceling current harmonics generated by diode-based three-phase rectifiers. For example, this type of transformer is used in an AC drive based on CHB, Figure 1a, because its power cell is composed of a three-phase rectifier based on diodes—DC voltage link—a single-phase inverter voltage source, as shown in Figure 1c. It is possible to obtain an AC input current with low THD (3.0%) with the above.

The multipulse transformer is bulky, expensive, and must be designed for a fixed value of the power cell to be fed and not allow the modularity of the power cell to be extended to the transformer.

This work proposes to use the THD minimization and harmonic cancellation scheme [28]. It is necessary to use AFE rectifiers and change the input transformer for a simpler design.

The proposed power cell contains a three-phase wye-wye transformer, a voltage source AFE rectifier, and the load is modeled by a resistor for simplicity. The power topology is shown in Figure 1b and is composed of three power cells, *n_c_* = 3, which feed one phase of the AC load side. In this case, the single-phase voltage source inverter is simplified in its modeling as a resistor for simplicity. Although the analysis is performed for *n_c_* = 3, the proposed method is not restricted to this number and can be extended to any number of power cells, *n_c_*.

The operating principle of the switching states of an AFE rectifier should be considered to be of the voltage source type, which indicates that in each leg of the rectifier, (the pairs of switches (s_1_–s_4_), (s_3_–s_6_), and (s_5_–s_2_)), no short circuit should be generated; as a consequence, these switches operate in a complementary way. Furthermore, only one switch per leg must be on, while the other must be off. In particular, due to the above condition, this rectifier has eight valid switching states, as shown in Table 2.

The mode of operation that an AFE rectifier has will depend on the direction of the *i_dc_* current. When *i_dc_* is positive, the converter operates as a rectifier. In particular, the control scheme makes a current flow from the AC side to the DC side. From this perspective, the converter behaves like a booster. Then, if *i_dc_* is negative, the converter operates as an inverter, flowing a current from the DC side to the AC side, operating as a buck converter. Besides, this AFE rectifier can operate in four quadrants: as a leading power factor rectifier, a lagging power factor rectifier, a leading power factor inverter, and a lagging power factor inverter.

For the consideration of the wye-wye transformer between the AFE rectifier and the AC power supply, it is preferred to obtain a model referred to as the secondary of the transformer, Figure 1d, due to the greater ease of formulating the FCS–MPC algorithm for the input current control of the AFE rectifier. In addition, in the modeling, the magnetization branch of the transformer is neglected.

## 3. Harmonic Minimization

**Figure 2:**
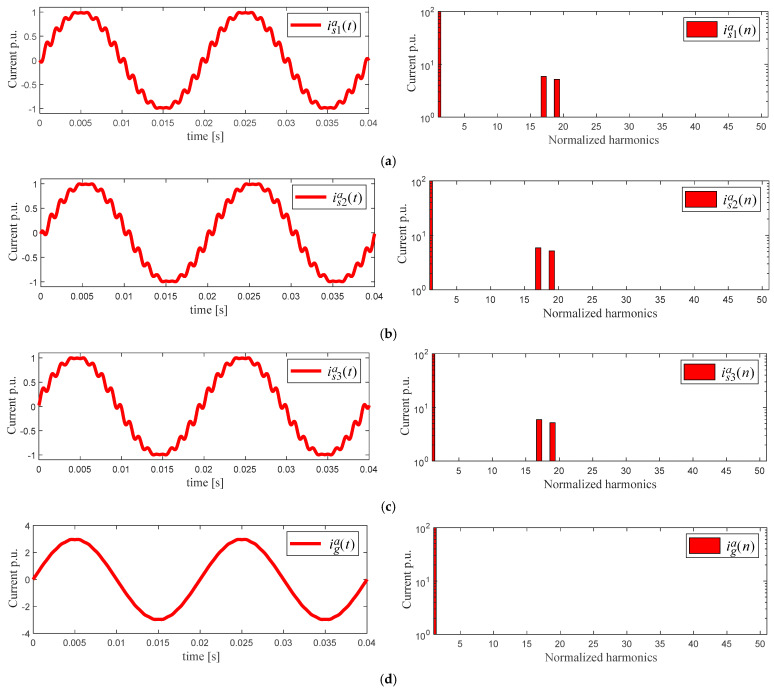
Input current references and harmonic content AFE rectifier for phase *a*. (**a**) AFE rectifier 1, (**b**) AFE rectifier 2, (**c**) AFE rectifier 3, (**d**) input current multi-cell AFE rectifier.

The input current reference on each AFE rectifier **i***_si_^abc^* is generated to emulate an 18th pulse diode input current, leading to the 17th and 19th dominant harmonics, Figure 2a–c. The objective is to obtain a defined spectrum in each power cell.

However, the 17th and 19th harmonics are not desired in the overall input current **i***_g_^abc^*. Thus, the input current reference in each power cell **i***_si_^abc^* has a phase-shift angle α at the fundamental frequency. This angle is calculated offline to obtain the minimum THD in the overall input current of the topology **i***_g_^abc^*. Indeed, the harmonics contained in **i***_si_^abc^* are minimized, and they practically do not appear in the overall input current of the multicell AFE rectifier **i***_g_^abc^*, as shown in Figure 2d.

The proposed THD minimization reported in [28] considers the fundamental components of the input currents in each power cell, where angle α is responsible for minimizing the harmonic content.

To compute the phase-shift angle α, it is necessary to characterize the current in the transformer secondary winding, which will be used as the input current reference in the AFE rectifiers. Thus, the input current of a multicell AFE rectifier in phase a is given by:

(1) $$i_{g}^{a}\left(t\right)=\frac{I}{N_{P}}\left[3\cos\left(\alpha \right)\sin\left(\omega t\right)-\frac{\sin\left(17\cdot \omega t\right)\left[\cos\left(\alpha \right)+2\cos\left(17\cdot \alpha \right)\right]}{17}-\frac{\sin\left(19\cdot \omega t\right)\left[\cos\left(\alpha \right)+2\cos\left(19\cdot \alpha \right)\right]}{19}\right]$$

The input current $i_{g}^{a}$ is defined in (1), where the phase is 0° owing to the desired unitary displacement factor concerning the input voltage $v_{g}^{a}$.

THD minimization of the overall input current in the multicell AFE rectifier is performed using the expression defined by,

(2) $$\mathrm{THD}\left(I\right)=\frac{\sqrt{\sum_{k=2}^{n\;=\;51}I_{k}^{2}}}{I_{1}}\times 100,$$

Thus, the THD expression (2) for the overall input current in the multicell AFE rectifier in phase a is given by,

(3) $$\mathrm{THD}\left(i_{g}^{a}\right)=\frac{\sqrt{{\left(\frac{\cos\left(\alpha \right)+2\cdot \cos\left(17\cdot \alpha \right)}{17}\right)}^{2}+{\left(\frac{\cos\left(\alpha \right)+2\cdot \cos\left(19\cdot \alpha \right)}{19}\right)}^{2}}}{3\cdot \cos\left(\alpha \right)}\times 100\%.$$

where (3) is used to minimize the THD of the overall input current in the multicell AFE rectifier. This minimizes the 17th and 19th harmonics by obtaining the optimum phase shift α.

On the other hand, this minimization is subject to the following constraint,

(4) $$0<\alpha \leq \frac{\pi }{2}.$$

Minimization is performed in MATLAB^®^ using the fmincom command, which minimizes nonlinear expressions considering the constraints. The result of the THD minimization was α = 6.671°, obtaining a THD of 0.561% in the overall input current in the multicell AFE rectifier **i***_g_^abc^*.

The results of the THD minimization are the waveforms shown in Figure 2a–c. These waveforms are used as references to control each power cell input current **i***_si_^abc^*. These current references have five aims: (i) to obtain a defined harmonic content in each power cell, improving a main drawback of the FCS–MPC, that is: spread harmonic spectrum, and (ii) due to the use of three power cells, the current harmonic compensation is performed to emulate an 18th pulses rectifier (6·*n_c_* = 18), getting a 0.561% THD in **i***_g_^abc^*, (iii) to allow the input current **i***_g_^abc^* generation with low THD, based on currents **i***_si_^abc^* with lower quality, (iv) to extend the conventional power cell module, including the input transformer, leaving the harmonic minimization to the control scheme, and (v) input transformers with a more straightforward design, with a K-Factor = 4.00 in both windings, which is less than the K-Factor = 9.00 in the secondary winding of the multipulse input transformer [29], and therefore, the construction of this wye-to-wye transformer is less bulky than conventional ones.

It can be seen in Figure 2d that **i***_g_^abc^* does not contain the 17th and 19th harmonics (or feature amplitudes lower than 1%) because α is calculated to minimize the harmonics of the input currents AFE rectifiers **i***_si_^abc^* Figure 2a–c, in each power cell.

Table 3 presents a performance comparison for the proposed THD minimization for *n_c_* = 3, 4, 5, 6, power cells. According to the Nyquist sampling criterion, in theory, it is possible to implement current references with a high sampling time, making it possible to implement them in the DSP. Obtaining the phase shift angle α varies depending on the *n_c_* power cells; however, the THD in the input current of the multicell AFE rectifier is less than 1%. Then, when comparing the losses of a multipulse transformer and a wye-wye transformer, a reduction in *F_HL-OSL_* greater than 25% and a reduction greater than 7% in *P_CU_* are achieved. Finally, despite a current profile with reduced harmonic content, the K-Factor = 4 for the wye-wye transformer for all values of *n_c_*. This is still a better condition than K-Factor = 9 for the secondary winding of the multipulse transformer. Details of loss analysis in transformers are reviewed in [28].

## 4. Control Scheme

**Figure 3:**
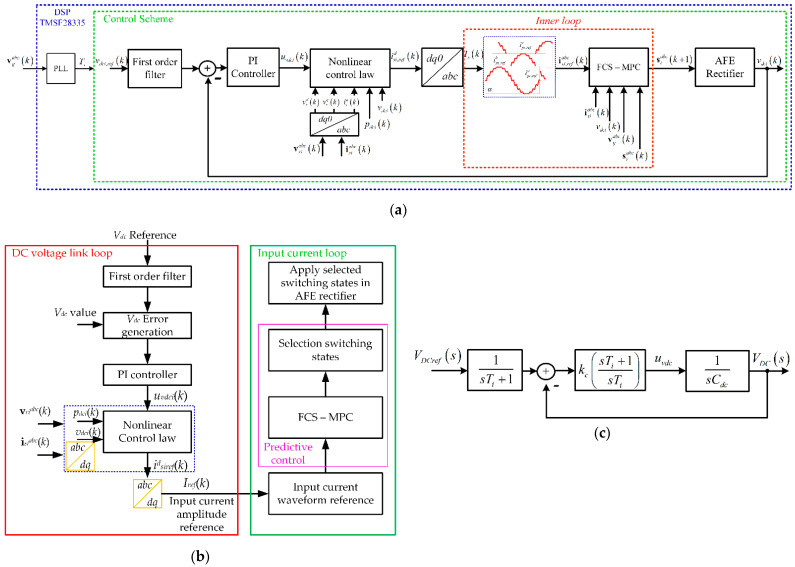
Control scheme in each power cell: (**a**) master-slave control scheme, (**b**) flow chart control scheme, (**c**) nonlinear control DC voltage with a filter.

The proposed control scheme is based on a dedicated master-slave scheme for each power cell, as shown in Figure 3a. The master loop (outer) controls the DC voltage *v_dc_* of the power cell, and the slave loop (inner) manages the input current in each AFE rectifier, Figure 3b. As can be seen in (7) and (9), any change in dc voltage will affect the input current of the AFE rectifier. This is independent of the type of load on the dc side. On the other hand, there is a dedicated control scheme since each power cell controls its variables independently, not depending on other power cells. Therefore, there is no hierarchical control of the variables, such as the control of the AC input current of the multicell AFE rectifier, and it sends signals to the power cells for the activation of the semiconductor devices or references to the control schemes of the power cells to manipulate the input current or DC voltage.

Correct synchronization with the AC grid is necessary for the proper operation of the control scheme and the THD minimization strategy [29,30].

### 4.1. Master Loop–DC Voltage Link

**Figure 4:**
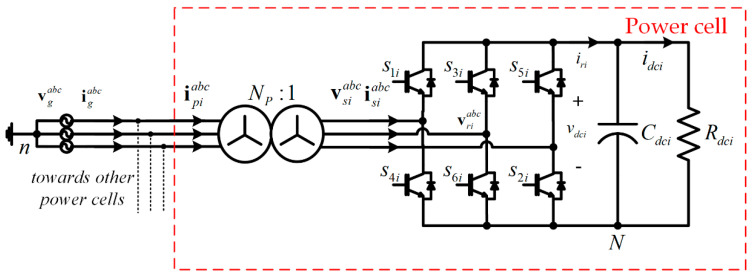
Power cell in multicell AFE Rectifier.

The DC voltage control is usually designed as a linear controller (PI), despite the AFE rectifier being a nonlinear system. Unfortunately, linear controllers can cause the system to become unstable, depending on the operating mode of the power converter. Therefore, a nonlinear control law is used in this work, which prevents the AFE rectifier from becoming unstable under the change of passive and regenerative modes in the load [24,25,26,27]. By performing a power balance in the AFE rectifier, as shown in Figure 4, we can write,

(5) $${\left[v_{ri}^{abc}\right]}^{T}\left[i_{si}^{abc}\right]=v_{dci}\;i_{ri}.$$

Thus, by applying the Park transform in (5) and considering a passive load, it follows that:

(6) $$\frac{3}{2}\left(v_{ri}^{d}i_{si}^{d}+v_{ri}^{q}i_{si}^{q}\right)=v_{dci}C_{dci}\frac{dv_{dci}}{dt}+\frac{v_{dci}^{2}}{R_{dci}}.$$

Then, after linearizing, the Transfer Function (T.F.) between *v_dci_* and *i^d^_si_* is:

(7) $$\frac{v_{dci}\left(s\right)}{i_{si}^{d}\left(s\right)}=\frac{3}{2}\frac{V_{r}^{d}R_{dc}}{V_{dc}}\frac{1}{sC_{dc}R_{dc}+2}$$

On the other hand, applying the Park transform in (5) and considering an active load, it is found:

(8) $$\frac{3}{2}\left(v_{ri}^{d}i_{si}^{d}+v_{ri}^{q}i_{si}^{q}\right)=v_{dci}C_{dci}\frac{dv_{dci}}{dt}+v_{dci}i_{dci}.$$

Then, after linearizing the T.F. between *v_dci_* and *i^d^_si_*, for an active load is,

(9) $$\frac{v_{dci}\left(s\right)}{i_{si}^{d}\left(s\right)}=\frac{3}{2}V_{r}^{d}\frac{1}{sC_{dc}V_{dc}+I_{dc}}.$$

Analyzing (7), the pole is located in a left half-plane *p* = −2/*C_dc_R_dc_*, but in (9), the pole is given by *p = −I_dc_*/*C_dc_V_dc_*, which depends on *V_dc_* and *C_dc_*, always positive, but *I_dc_* depends on the operation mode of the AFE rectifier, in regeneration mode *I_dc_* < 0; thus, (9) is unstable even if a PI controller is used in a DC voltage control loop.

The Equation (6) shows the model for a passive load and (8) an active load, exhibiting both representations of the instantaneous power provided to the load, where this power can be considered *p_dci_*. Both expressions are valid if a resistor drains instantaneous power. In the same way, the components of the input currents of the AFE rectifier can be replaced by the references owing to the fast response of the inner control loop, the aim of which is the stability in the DC voltage loop. Therefore, (6) and (8) can be written as,

(10) $$v_{dci}C_{dci}\frac{dv_{dci}}{dt}=\frac{3}{2}\left(v_{ri}^{d}i_{siref}^{d}+v_{ri}^{q}i_{siref}^{q}\right)-p_{dci}.$$

Then, if the right side of (10) is chosen to be equal to a new input named *u_vdci_* times *v_dci_*, then

(11) $$v_{dci}C_{dci}\frac{dv_{dci}}{dt}=u_{vdci}v_{dci},$$

that defines the nonlinear control law as:

(12) $$i_{siref}^{d}=\frac{\frac{2}{3}\left(u_{vdci}v_{dci}+p_{dci}\right)-v_{ri}^{q}i_{siref}^{q}}{v_{ri}^{d}}.$$

Thus (11), the new representation indicates the DC voltage behavior independent of the load type and operating mode. Indeed, the following linear T.F. between the controlled and manipulated variable is obtained:

(13) $$\frac{v_{dci}}{u_{vdci}}=\frac{1}{sC_{dci}}.$$

Therefore, using a PI-type linear controller ensures DC voltage regulation, dynamic response, and stable operation. Considering that the inner loop is made at least ten times faster than the outer loop, the closed-loop T.F. for the DC link becomes,

(14) $$\frac{V_{dc}\left(s\right)}{V_{dcref}\left(s\right)}=\frac{k_{c}\left(sT_{i}+1\right)}{s^{2}T_{i}C_{dc}+sk_{c}T_{i}+k_{c}}.$$

As can be seen, in (14) it does not fit the standard structure of a second-order T.F. because of the zero located at −1/*T_i_*. Therefore, a first-order filter is applied to the DC voltage reference to cancel out the zero in (14). The block diagram that includes the filter is shown in Figure 3c, and the resulting closed-loop T.F. is:

(15) $$\frac{V_{dc}\left(s\right)}{V_{dcref}\left(s\right)}=\frac{k_{c}\left(sT_{i}+1\right)}{s^{2}T_{i}C_{dc}+sk_{c}T_{i}+k_{c}}\frac{1}{\left(sT_{i}+1\right)}=\frac{\frac{k_{c}}{T_{i}C_{dc}}}{s^{2}+s\frac{k_{c}}{C_{dc}}+\frac{k_{c}}{T_{i}C_{dc}}}.$$

It can be seen that in (15), it has the same structure as a standard second-order T.F.. Therefore, it is possible to determine the PI controller parameters depending on the desired response (step input in this case): the overshoot ξ, settling time *t_s_*, and settlement band *δ*.

The settlement time is given by:

(16) $$t_{s}=\ln\left[\frac{1}{\delta \sqrt{1-\xi^{2}}}\right]\frac{1}{\xi \omega_{n}}.$$

Then, we compare term by term in (15) with a standard second-order T.F., to obtain the parameters of the linear controller and the first-order filter:

(17) $$k_{c}=\frac{2C_{dc}}{t_{s}}\ln\left[\frac{1}{\delta \sqrt{1-\xi^{2}}}\right].$$

(18) $$T_{i}=\frac{2t_{s}\xi^{2}}{\ln\left[\frac{1}{\delta \sqrt{1-\xi^{2}}}\right]}.$$

It is observed from (17) and (18) that the controller parameters are obtained according to the system parameters and desired step response.

Then, choosing the response parameters with a settling time of *t_s_* = 300 ms, an overshoot of 5% (*ξ* = 0.707), and the settling band *δ* = 2%, and *C_dc_* = 4.7 mF; it is obtained that the PI controller parameters are *k_c_* = 0.13 y *T_i_* = 0.07.

### 4.2. Slave Loop–Input Current

FCS–MPC manages the input current control of each power cell, where the input current references follow the waveforms depicted in Figure 2a–c, and are explained in Section 3. The primary purpose of this input current control loop is to replace the input multipulse transformer commonly used in AC drives based on CHB converters with a simpler input wye-wye transformer. This is because the proposed current control scheme and the THD minimization scheme perform the minimization of current harmonics.

The second objective of this control scheme is to concentrate the input current harmonic spectrum of each AFE rectifier. Thus, the main disadvantage of the FCS–MPC is the spread harmonic content, which in turn avoids resonance problems on the passive filters. Finally, a significant consequence of this control loop is the integration of the input transformer into the power cell, enhancing the modularity of the proposal.

On the other hand, the choice of the FCS–MPC for input current control is due to the following advantages: (i) rapid response to changes in references, (ii) natural use of the discrete nature of power converters, and (iii) easy inclusion of nonlinearities and operating restrictions of the converter.

To design the FCS–MPC, it is necessary to obtain the input current model of each power cell, as shown in Figure 4. Then, the model can be defined in continuous time and discretized using the Euler forward approximation, resulting in:

(19) $$i_{pi}^{abc}\left(k+1\right)=\left[1-\frac{\left(R_{p}+N_{P}^{2}R_{s}\right)}{\left(L_{p}+N_{P}^{2}L_{s}\right)}T_{s}\right]i_{pi}^{abc}\left(k\right)+\left[v_{p}^{abc}\left(k\right)-N_{P}Ms_{i}^{abc}\left(k\right)v_{DCi}\left(k\right)\right]\frac{N_{P}T_{s}}{\left(L_{p}+N_{P}^{2}L_{s}\right)}.$$

Owing to the use of a wye-wye input transformer, the power cell model is referred to as the secondary winding of the transformer, as shown in Figure 1d, where the magnetic branch has been neglected. Using (19), it is possible to predict the future behavior of the input current in each power cell. Finally, considering that the compensation delay for calculation (19) is rewritten at instant *k* + 2, considering $v_{p}^{abc}\left(k\right)\approx v_{p}^{abc}\left(k+1\right)$ and $v_{dc}\left(k\right)\approx v_{dc}\left(k+1\right)$:

(20) $$i_{pi}^{abc}\left(k+2\right)=\left[1-\frac{\left(R_{p}+N_{P}^{2}R_{s}\right)}{\left(L_{p}+N_{P}^{2}L_{s}\right)}T_{s}\right]i_{pi}^{abc}\left(k+1\right)+\left[v_{p}^{abc}\left(k\right)-N_{P}Ms_{i}^{abc}\left(k+1\right)v_{DCi}\left(k\right)\right]\frac{N_{P}T_{s}}{\left(L_{p}+N_{P}^{2}L_{s}\right)}.$$

Once the current reference is generated, it is compared with the estimated current of (20), where all the AFE rectifier possible states are tested (as shown in Table 2), choosing the one that minimizes the cost function defined as:

(21) $$g_{i}\left(k+2\right)=\sum_{j\;=\;a}^{c}{\left|i_{si}^{j^{*}}\left(k+2\right)-i_{si}^{j}\left(k+2\right)\right|}^{2},$$

The cost function defined in (21) controls the input current for each *i*-th AFE rectifier. Thus, this ensures a low THD and unitary displacement power factor in the total input current owing to the tracking of the input current references of the AFE rectifiers. Nevertheless, an arbitrary power factor can be imposed to compensate for the reactive power if required, as long as it is inside the operating region.

## 5. Experimental Results

**Figure 5:**
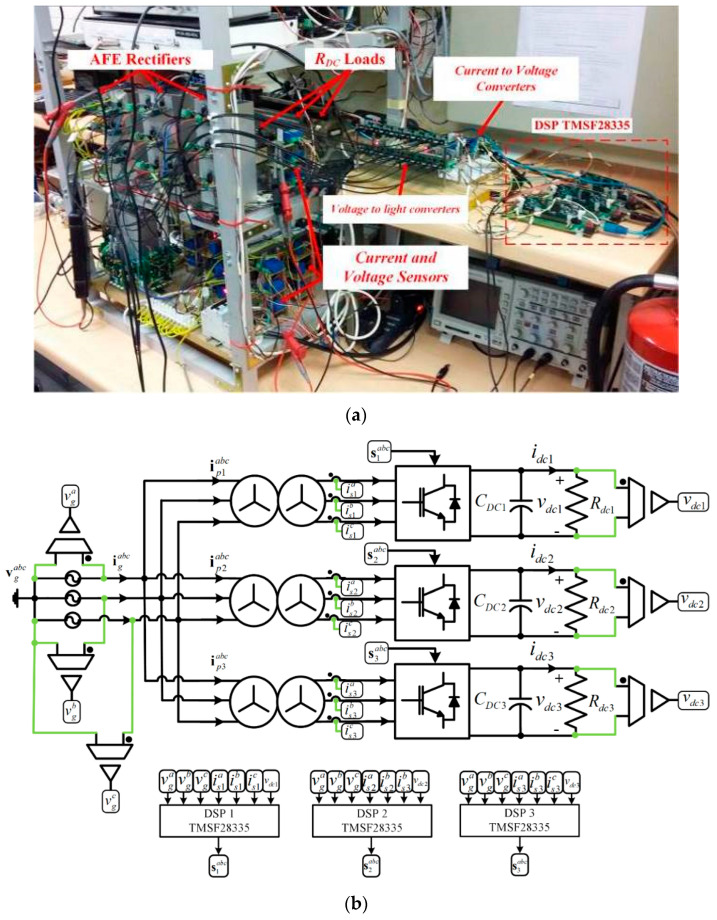
Experimental setup, (**a**) low power prototype multicell AFE rectifier, (**b**) Input/output signals block diagram of experimental setup.

A three-power-cell-based experimental prototype was assembled to test the proposed control scheme. The prototype is depicted in Figure 5a and uses three digital DSP TMS320F28335 boards, where each board controls an individual AFE rectifier in a dedicated manner. Details of the input and output signals of the DSP are shown in Figure 5b.

**Figure 6:**
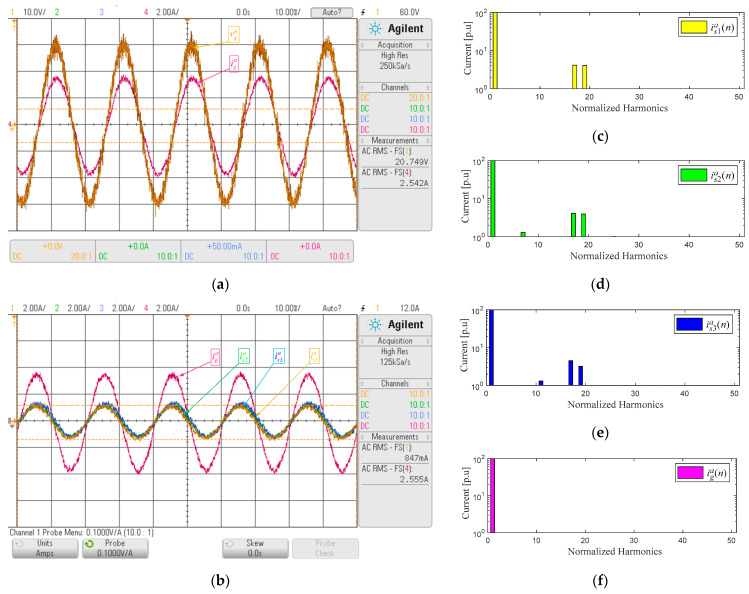
Key waveforms input current control: (**a**) input voltage (yellow) and current (magenta) v_p_^a^ and i_g_^a^, (**b**) input current phase a i_s1_^a^ (yellow), i_s2_^a^ (green), i_s3_^a^ (blue) and i_g_^a^ (magenta), (**c**) harmonic spectrum i_s1_^a^, (**d**) harmonic spectrum i_s2_^a^, (**e**) harmonic spectrum i_s3_^a^, (**f**) harmonic spectrum i_g_^a^.

The experimental parameters are listed in Table 4. Key waveforms for input current control in the steady-state are presented in Figure 6 to show the performance of the proposed control scheme to equalize the input current and generate the desired frequency spectrum. Figure 6a shows the overall AC current, and AC input voltage, Figure 6b shows the input current in each power cell for phase *a*, while the spectrum of each current is presented in Figure 6c–e, and Figure 6f shows the spectrum of the overall AC input current. It is appreciated that the power cell input currents have distortion due to the 17th and 19th harmonics injected by the control scheme, leading to a band between the fundamental and 17th harmonics. However, these harmonics were not observed in the overall current. This is due to the angle α, which is calculated such that these harmonics do not appear in the AC current grid. However, in Figure 6d,e, some low-frequency harmonics are present because of the number of points per period (0.02/*T_s_* = 360), which allows a resolution of 1° and limits the precise implementation of the α angle.

Figure 6a shows that the input voltage *v_g_^a^* and current *i_g_^a^* of the multicell AFE rectifier are in phase, leading to a unitary displacement power factor, and the input current does not contain the 17th and 19th harmonics, Figure 6f. This is because of the correct calculation and implementation of the angle α using a PLL [29,30]. This calculation was performed to minimize the 17th and 19th harmonics present in each input current of the AFE rectifier. Getting a 1.87% THD in the input current of the multicell rectifier AFE, thus the multipulse transformer could be replaced by an array of simpler transformers (wye-wye), extending the power cell modularity up to the input transformer level.

Independent of the input current control of each AFE rectifier, the DC voltage control was nonlinear. Thus, a nonlinear control strategy is recommended to provide stability in any operating regime in which PI controllers are not attainable. Using (12), it is possible to establish a linear relationship between the controller output and the DC voltage through the given control law.

**Figure 7:**
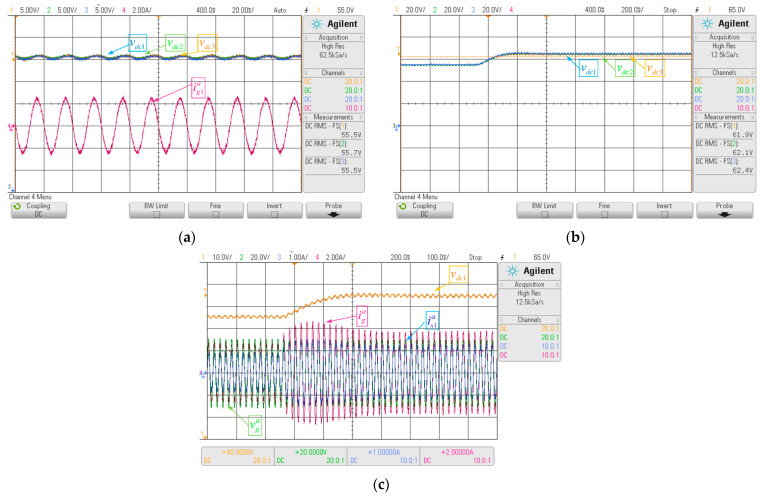
Key waveforms DC voltage control: (**a**) steady-state behavior, (**b**) dynamic behavior with step input in DC voltage reference, (**c**) change reference in input current loop due to change reference in DC voltage loop.

Next, the DC voltage control behavior, steady-state, and dynamic state were reviewed. Figure 7a shows the correct operation of the DC voltage controller in steady-state, where there is no error between the DC voltage and its reference, with the ripple of the DC voltages being less than 2%. This ripple is due to the number of points used per period (360), generating current *i_ri_* with harmonics at the fundamental frequency, which is reflected in the DC voltage.

The dynamic behavior of the controller is shown in Figure 7b. The system was subjected to a step-change of 18% (10 V), which goes from 55 V to 65 V. The DC voltages respond to the change imposed by the DC voltage references, where it is seen that the overshoot is less than 5% and the settling time is 300 ms. For this test, the parameters of the PI linear controller are *k_c_* = 0.13 and *T_i_* = 0.07.

Finally, the input current behavior of the multicell AFE rectifier under step-type changes was analyzed. Owing to the nature of the master-slave control scheme, any change in the DC voltage affects the input current of the AFE rectifiers. For example, the above is shown in Figure 7c, where the increase in the DC voltage in AFE rectifier 1 increases the input current of power cell 1 and, as a result, it increases the overall input current of the rectifier AFE multicell. Furthermore, the input voltage and input current are in phase, leading to a unitary displacement power factor that is imposed by the harmonic minimization strategy on the input current of the multicell AFE rectifier.

## 6. Conclusions

A control scheme is presented based on a nonlinear control strategy, and an FCS–MPC is applied to a multicell AFE rectifier. As a result, it is possible to minimize the input current harmonics of AFE rectifiers through the FCS–MPC while maintaining a unitary displacement power factor. Mainly, the THD minimization based on the calculation of a phase-shift angle α fulfills four objectives: (i) it fixes the input current spectrum of the AFE rectifiers; (ii) owing to the use of three power cells, the current harmonic compensation is made to emulate a three-phase diode rectifier of 18 pulses, but achieves a 1.87 % THD in the AC grid input current, which is lower than the recommended by the Standards IEEE 519 and IEEE 1547 [31,32]; (iii) the fact of resulting in modular equipment allows the generation of an input current ***i****_g_^abc^* with low THD out of the currents ***i****_si_^abc^* with lower quality; and (iv) the replacing of the bulky and expensive multipulse input transformer with an input transformer with a more straightforward design and lower K-factor. Furthermore, the overall input current of the multicell AFE rectifier was 1.87% THD, with a unitary displacement factor.

The THD minimization can be implemented for *n_c_* = 4, 5, and 6, achieving a reduced THD <1% in the overall input current of the multicell AFE rectifier. In addition, it has been possible to reduce the transformer losses, particularly *F_HL-OSL_* < 26% and *P_CU_* <7.2%, while the K-Factor is the same for all *n_c_* values, thus reducing the design complexity in comparison to the multipulse transformer.

The nonlinear control scheme with filtered reference obtains a stable regulation—for all operating conditions—of the DC link voltage of the power cell, with a ripple of approximately 2%. As a result, the DC voltage dynamic behavior is managed correctly with an overshoot of less than 5% and a settling time of 300 ms. In addition, the filter inclusion on the DC voltage references smooths the current references, avoiding more significant load impacts at the AC grid. The experimental tests demonstrated the correct performance of the proposed scheme.

## Figures and Tables

**Table 2:** Switching states AFE rectifier.

State	1	2	3	4	5	6	7	8
$s_{1}$	0	1	1	0	0	0	1	1
$s_{3}$	0	0	1	1	1	0	0	1
$s_{5}$	0	0	0	0	1	1	1	1

**Table 3:** Comparison between different number of cells.

n_c_	h	Minimum *T_s_*	α	THD i*_g_^a^*	I_rms,sec_ (p.u.)	Reduction F_HL-OSL_	Reduction P_CU_	K-Factor
3	17, 19	526 [μS]	6.671	0.561 %	1.003	26.75%	7.2%	4.00
4	23, 25	400 [μS]	3.772	0.272 %	1.002	27.34%	7.4%	4.00
5	29, 31	323 [μS]	4.863	0.137 %	1.001	28.49%	7.6%	4.00
6	35, 37	270 [μS]	3.341	0.132 %	1.001	28.90%	7.6%	4.00

**Table 4:** Experimental Parameters.

Symbol	Variable	Value
*v_g_*	Phase voltage	31.1 [V]
*f*	Network frequency	50 [Hz]
*α*	phase shift angle	6.671°
*R_p_*, *R_s_*	Transformer primary and secondary resistances	3 [Ω]
*L_p_*, *L_s_*	Transformer primary and secondary inductances	6 [mH]
*N_P_*	Turns ratio	1
*R_dc_*	DC load resistance	89 [Ω]
*C_dc_*	DC link capacitor	4.7 [mF]
*V_dc_*	DC link voltage	55 [V]
*k_c_*	Proportional gain	0.13
*T_i_*	Integral time	0.07
*T_s_*	Sampling time	55.56 [µs]

## Data Availability

Not applicable.
